# Impact of Apparatus Orientation and Gravity in Electrospinning—A Review of Empirical Evidence

**DOI:** 10.3390/polym12112448

**Published:** 2020-10-22

**Authors:** Sinduja Suresh, Alexander Becker, Birgit Glasmacher

**Affiliations:** 1 Institute for Multiphase Processes (IMP), Leibniz University Hannover, 30823 Garbsen, Hannover, Germany; glasmacher@imp.uni-hannover.de; 2 Lower Saxony Centre for Biomedical Engineering, Implant Research and Development (NIFE), 30625 Hannover, Germany; 3 Hannover Medical School (MHH), 30625 Hannover, Germany

**Keywords:** electrospinning, apparatus, orientation, gravity, electric field, angle, vertical, horizontal, fibre, jet

## Abstract

Electrospinning is a versatile fibre fabrication method with applications from textile to tissue engineering. Despite the appearance that the influencing parameters of electrospinning are fully understood, the effect of setup orientation has not been thoroughly investigated. With current burgeoning interest in modified and specialised electrospinning apparatus, it is timely to review the impact of this seldom-considered parameter. Apparatus configuration plays a major role in the morphology of the final product. The primary difference between spinning setups is the degree to which the electrical force and gravitational force contribute. Since gravity is much lower in magnitude when compared with the electrostatic force, it is thought to have no significant effect on the spinning process. But the shape of the Taylor cone, jet trajectory, fibre diameter, fibre diameter distribution, and overall spinning efficiency are all influenced by it. In this review paper, we discuss all these developments and more. Furthermore, because many research groups build their own electrospinning apparatus, it would be prudent to consider this aspect as particular orientations are more suitable for certain applications.

## 1. Introduction

The discovery of electrostatic attraction by William Gilbert in the 16th century [1] sparked worldwide interest in the distortion of droplets under electrostatic force and electrically driven jets, culminating in the development of a process now known as electrospinning. In the recent past, electrospinning has emerged as the preferred method for the fabrication of nano- or microfibrous structures in textile and tissue engineering applications due to its versatility and ease of operation.

Electrospinning (or electrostatic fibre spinning) is a process that involves the stretching of a viscoelastic melt/solution into a fibre of nano- or micrometre dimensions under the driving influence of an externally applied electrostatic force. The apparatus is set up with a syringe containing a polymer solution/melt fixed at a certain distance from a grounded collector. The solution/melt is extruded through the needle tip [2,3,4,5,6].

**Figure 1:**
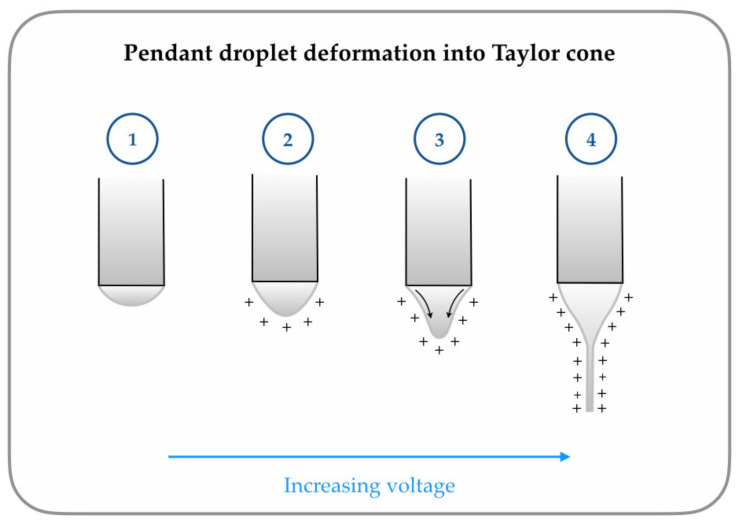
Droplet deformation modes of polymer solution/melt in electrospinning: Mode 1—there is no droplet deformation when there is no voltage. Mode 2—charges accumulate around the droplet on the application of low voltage. Mode 3—Coulombic repulsion overpowers surface tension, and the droplet starts to deform into a Taylor cone. Mode 4—at high voltages, the Taylor cone enters “jetting mode,” and a fibre is extruded from the tip.

The polymer droplet undergoes four stages of deformation under the application of high voltage, culminating in the formation of a fibre jet (Figure 1). When voltage is applied, charged ions accumulate around the polymer droplet. Increasing the voltage allows for the Coulombic repulsion between the ions to overcome the force of surface tension and deform the polymer droplet into what is known as the “Taylor cone.” At a critical voltage, a fine fibre jet (in either the nano- or micrometre range depending on the operating parameters) is extruded from the tip of the Taylor cone towards the grounded collector. The solvent (if present) evaporates during the time the fibre reaches the collector, resulting in the formation of a dry dense fibrous mesh [6,7,8,9].

The process of electrospinning is influenced by a number of tuneable variables commonly classed as process (applied voltage, feed/flow rate, tip-to-collector distance (TCD)), solution (concentration, conductivity, viscosity, surface tension, polymer molecular weight), ambient (temperature, humidity), and other variables. All these variables and their effects on the process and final electrospun product have been discussed in great detail throughout the literature (Table 1). However, there is one parameter that is seldom considered—the configuration of the apparatus itself.

**Figure 2:**
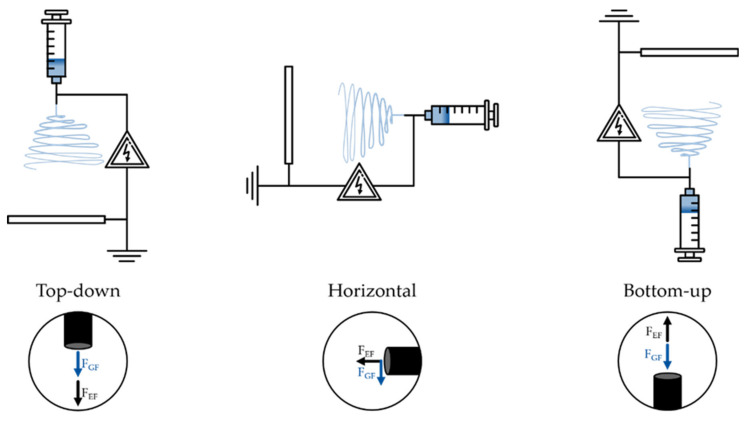
Schematic of the different electrospinning setups (GF—gravity, EF—electric field).

There are three typical electrospinning setups [27,28,29,30,31]—horizontal, top-down, and bottom-up. In the horizontal system, the needle tip and collector are arranged in such a way that electrospinning happens parallel to the ground. Gravity, in this case, acts perpendicular to the electrical forces. In the top-down system, the fibre formation occurs in the direction of gravity. In the bottom-up system, the fibre formation occurs against the direction of gravity (Figure 2).

To date, there are a startlingly low number of publications that even address the issue of apparatus orientation. Within this limited literature, the relative importance of gravity in different setups has been subject of considerable debate. Most research into electrospinning disregards the effect of gravity since its magnitude is negligible compared with other driving forces involved in electrospinning, and only very few analyses have taken gravity into account. However, experimental research into electrospinning setups contains evidence that the setup orientation and the effect of gravity have an influence on the final electrospun product. Until now, a clear basis of variability in differently electrospun (top-down, bottom-up, or horizontal) products has not yet been established. However, there have been some accounts of the influence of setup orientation on the electrospinning process and its outputs.

To the best of our knowledge, there is currently no review on experimental data obtained from studies comparing horizontal and vertical electrospinning. Therefore, this review aims to provide a concise but exhaustive overview on experimental insights into electrospinning in different setups. We will answer these questions: Is the orientation of the electrospinning setup a parameter to seriously consider when producing scaffolds for tissue engineering? Is further research into this area warranted? With basic research in the past, current endeavours into electrospinning are increasingly focusing on modified electrospinning setups for specialised scaffolds. At a time of significant interest in this area, we present a state-of-the-art review and hope it will fuel further research in this field.

## 2. State of the Art—Impact of Apparatus Orientation in Electrospinning

What we know about the impact of setup orientation on the electrospinning process is largely based on empirical data that investigate the formation of Taylor cones, morphology of jet trajectories, fibre deposition tendencies, and overall efficiency of the experimental approach. In the following subsections, qualitative and quantitative experiment-based research investigating electrospinning in different setup orientations has been detailed.

### 2.1. Gravity-Affected Taylor Cones and Fibre Jets

The Taylor cone is influenced by a multitude of factors—flow rate [32,33,34], conductivity [32], nozzle diameter [32] and geometry (in coaxial spinning) [35], viscosity [32], polymer used [36], surface charge [37], charge density [38], applied voltage [33,39], and solvent [40]. Additionally, an interesting paper has investigated the effect of different setup orientations on the half-angle of the Taylor cone. Yarin et al. [41] noted a smaller half-angle for top-down electrospinning (31°, 26°) than for bottom-up electrospinning (37.5°, 30.5°).

**Figure 3:**
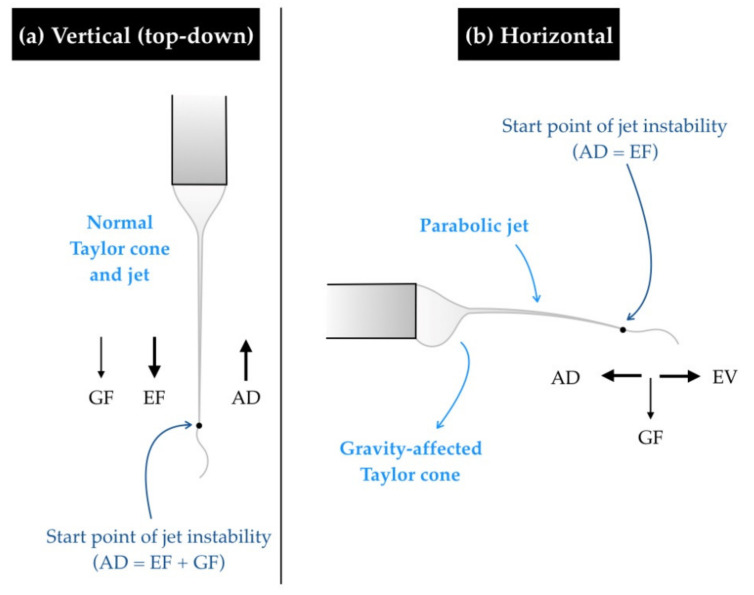
Taylor cone distortion and parabolic jet in horizontal electrospinning. (GF—gravity, EF—electric field, AD—aerodynamic drag). (**a**) The Taylor cone is undisturbed in the vertical orientation because GF and EF act in the same direction (top-down). (**b**) Gravity-affected Taylor cones and parabolic jets, as depicted, are commonly observed in the horizontal orientation. Here, GF and EF act perpendicular to each other.

One of the most obvious and immediately observable differences between horizontal and vertical spinning is the shape of the extruded polymer droplet and subsequently formed Taylor cone. In vertical electrospinning, the pendant drop is perfectly spherical, and the Taylor cone forms at the centre of the needle tip. However, in the horizontal system, the polymer droplet and subsequent Taylor cone are affected by gravity and tend to sag in the vertical direction (Figure 3). Gravity-affected Taylor cones have been reported in several studies. It has been noted that a tip-to-collector distance of more than 10 cm in a horizontal spinning setup can cause the effect of gravity on the fluid jet to become significant. This can in turn reduce effective yield because the fibre jet is dragged towards the bottom of the collector and can sometimes even be deposited below the collector [42].

The jet trajectory in the vertical system is straight (i.e., the jet travels directly from the needle tip to the collector in a straight line). Specifically, in the bottom-up configuration, gravity plays a major role in jet initiation [43] and seems to allow for more jet stability [44]. In contrast, horizontal systems show parabolic jet paths. A detailed account of the occurrence of a gravity-affected Taylor cone and parabolic jet path in a horizontal setup was presented by Suresh [28] in the electrospinning of a poly-ε-caprolactone (PCL)/gelatin blend.

In 2012, Rodoplu and Mutlu [45] conducted a large parametric study of polyvinyl alcohol (PVA) electrospinning, part of which dealt with the effect of setup orientation (horizontal and top-down). They stated that gravity seems to have no effect on the final product because its magnitude is negligible compared with the electric field. However, there was evidence that gravity influences the actual spinning itself. For instance, the polymer droplet shape (and Taylor cone, on the application of voltage) is affected by gravity, leading to a difference in spinning parameters in the two setups. They also explained that, in the horizontal setup, the fibre jet travels a parabolic path to the collector regardless of how the Taylor cone is oriented, and the fibres are collected towards the bottom of the collector. It is plausible that some fibres were lost below the collector during this electrospinning process.

Khenoussi et al. [46] considered the apparatus orientation when designing an electrospinning device for their experiments. They made a conscious decision to manufacture a top-down system because their first prototype, a horizontal system, showed gravity-affected jet trajectories. They considered this an imperfect setup. Since the effect of parabolic jets is not extensively studied, it is understandable that the group chose a top-down system in the interest of preserving the standardisation of their experiments.

Interestingly, the occurrence of gravity-affected jets in horizontal setups also means that the final product tends to have less artefacts, as drips and errant fibre bursts do not reach the collector due to their weight. Top-down vertical systems tend to produce slightly more imperfect electrospun products as these artefacts have nowhere to go but down to the collector. It has been noted that researchers sometimes prefer the horizontal system [28,47,48] or the bottom-up system [44,49] to the top-down system for this specific reason, especially when the electrospinning process is not fully stable.

### 2.2. Fibre Diameter, Fibre Diameter Distribution, and Fibre Mat Homogeneity

In 2009, Yang et al. [27] compared fibre diameters and fibre diameter distributions of electrospun polyvinylidene difluoride (PVDF) for a range of voltages in all three setup orientations—dubbed as shaft type (top-down), horizontal type, and converse type (bottom-up). They observed that the mean fibre diameter was lowest in the shaft type, medium in the horizontal type, and highest in the converse type. They also observed that the fibre diameter distribution was largest in the shaft type, medium in the horizontal type, and smallest in the converse type. The study finally concluded that the difference in fibre properties was due to gravity working with/strengthening (shaft), not influencing (horizontal) or working against/weakening (converse), the electric field. The bigger the combined resultant force of gravity and the electric field, the thinner the fibre and the larger the field disturbances, and vice versa. 

More recently, with the advent of melt electrospinning and melt electrospinning writing (MEW), Wunner et al. [50] notably researched the effect of gravity on MEW of PCL conducted in all three setup orientations. In contrast to the results reported by Yang et al., the average fibre diameter was seen to reduce from top-down to horizontal to bottom-up orientation in their account. Similarly, the results of the studies conducted by Khenoussi et al. are contradictory to what Yang et al. put forth. They claimed that the top-down system used in their experiments improved the homogeneity of the nonwoven mat when compared with the horizontal system.

In the study by Faissal et al. [44] (where they experimentally validated bottom-up electrospinning), gravity was heavily cited as the reason for increased stretching of the jet and consequently thinner fibres. In addition, they explained that Rayleigh and whipping instabilities are eliminated because the gravitational and electrostatic forces acting on the jet act in opposing directions [51]. 

### 2.3. Fibre Alignment

To our knowledge, there is only one prominent study in the literature till date relating apparatus setup, gravity, and fibre alignment. Pan et al. [52] examined the differences in polyacrylonitrile (PAN) nanofibre alignment when electrospun in either the horizontal or top-down orientation. The aligned nanofibres were obtained using two plate electrodes (gap spinning). They demonstrated that although top-down vertical and horizontal spinning produced similar fibre alignment between the two electrodes, top-down spinning had a much higher angle distribution than the horizontal type. They claimed that this is most likely due to the effect of gravity. Eventually, it was concluded that horizontal gap spinning produces a more optimal parallel fibre-aligned product compared with top-down gap spinning.

### 2.4. Mass and Density of Collected Electrospun Fibres

It is known that adjusting certain parameters like applied voltage, solution conductivity, and flow rate can change the diameter of the extruded jet and by extension the resulting mass of deposited fibres. But how does the setup orientation influence this? 

In general, the rate of fibre mass deposition is directly dependent on the charge density at the Taylor cone. According to Stranger et al. [53], an increase in charge density at the Taylor cone causes the jet to be drawn from a smaller effective area or “virtual orifice.” This in turn results in a smaller jet diameter and a lower rate of fibre mass deposition. 

However, assuming that the charge density in a top-down and horizontal system is the same, the rate of fibre mass deposition is also influenced by another factor—jet velocity. According to Taylor’s commentary on the electrospinning process [6], jet velocity in the horizontal system is lower than in the top-down system because the only driving force of the jet is the electric field. In the top-down system, the jet is driven towards the collector not only by the electric field but also by gravity. This results in a slightly higher jet velocity and consequently a higher rate of fibre deposition. This means that at the end of a specified time period, assuming all other parameters are constant, a vertically (top-down) spun polymer would have more fibres on the collector than its horizontally spun and bottom-up spun counterparts.

In the study conducted by Pan et al. [52] (mentioned in Section 2.3), they pointed out that the number of fibres (or rather the density of the fibre mesh) collected at the end of the spinning time was higher in the top-down system than in the horizontal system. They stated that gravity-driven jets were responsible for higher speeds of fibre collection and therefore resulted in a higher mass of collected fibres.

In a similar vein, Tun et al. [54] characterised the electrospinning of TiO_2_, albeit in vacuum. This is an interesting study because it shows the effect of gravity on the spinning process when other environmental variables have been removed (i.e., the process in both orientations was carried out in a glass tube in an evacuated chamber). They also noted that the density and number of electrospun fibres were much more in the top-down system than in the horizontal system and claimed it was due to the effect of gravity.

### 2.5. Intermediate Angles for Spinning

The above subsections give an idea of how an electrospinning system behaves in a horizontal or vertical setup. But to what extent do these effects appear (or disappear) when we consider an intermediate angle?

Zargham et al. [34] electrospun nylon 6 at an unusual angle of 45° from a horizontal baseline, on which the collector was placed. At a low flow rate of 0.5 mL/hr, the electrospinning was stable with very few unseen droplets on the collector. However, at higher flow rates of 1 and 1.5 mL/hr, they observed an increase in the effect of the gravitational force, resulting in distorted Taylor cones and more droplets being formed. Finally, at these rates, an electrospray phenomenon was observed where the electric field was unable to draw the fluid into a proper jet. They reported unstable spinning, which affected the mean droplet size and the distribution of droplet sizes.

Had the experiment been conducted in a top-down fashion, we would still see a pulsating Taylor cone at high flow rates but perhaps no electrospray phenomenon. The fibres would have a large size distribution, and the final product would have ribbonlike structures due to insufficient solvent evaporation. Had the experiment been conducted in a horizontal setup, the effects mentioned would have been exacerbated, resulting in an electrospray at lower flow rates than expected, especially considering that the tip-to-collector distance they used was 15 cm.

### 2.6. Melt Electrospinning

Just like solution electrospinning, melt electrospinning can be done in all three traditional configurations. It is important to note that it is not possible to use a transfusion line to transport the polymer melt from the plunger to the needle tip as the polymer will cool and solidify. Therefore, the plunger system has to be in line with the needle and connected directly. In the vertical configuration, such setup can be difficult to construct and significantly increase the height of the electrospinning device [55]. Arranged horizontally [56,57], this issue is somewhat mitigated, and the length of the device can be adjusted easily on a laboratory bench [55]. However, there are certain experiments that warrant a vertical setup, such as direct in vitro electrospinning. Liquids are used during cell culture, and it is not feasible to mount these dishes sideways without spills and sterility compromises [58].

There have been conflicting reports on whether gravity is really a factor in melt electrospinning or not. While some studies acknowledge that the gravitational force plays a part in the spinning process [49,59,60,61], others believe that it is eclipsed by the electrostatic force [62,63,64,65].

Experimentalists doing bottom-up electrospinning typically report larger fibre diameters because the fibre has to overcome the gravitational force and reach the collector [59]. In another notable study with top-down electrospinning where the researchers developed a “printability number” for electrospinning, gravity was considered one of the downward pulling forces that help to overcome the viscous and elastic stresses applied to the polymer melt [61].

In the multiparametric study mentioned in Section 2.2, Wunner et al. [50] concluded that at low flow rates, melt electrospinning can be conducted without variability at any orientation. However, at high flow rates, gravity affects the Taylor cone more in horizontal and bottom-up electrospinning than in top-down spinning, resulting in a pulsating Taylor cone and subsequent distortion of the intended scaffold architecture [50,66] in these cases. By keeping the flow rate small, and therefore the effect of gravity, the same group was able to upscale the melt electrospinning process. With an innovative apparatus design, they were able to horizontally spin on both sides of the collector and produce large scaffolds [67]. Therefore, they acknowledge that gravity can affect the electrospinning, but it is only an issue when accompanied by high flow rates.

As a side note, we would like to mention the effect of gravity in other aspects of melt electrospinning not directly related to the spinning process itself. It is commonly seen in multilayer melt electrospinning (and other additive manufacturing methods) that fibres spun in lattice formations tend to sometimes deform/sag in the direction of gravity between intersection points. This could be caused by either a slow cooling rate of the polymer, large distances between intersection points, or a combination of the two [65,68].

### 2.7. Coaxial and Blend Electrospinning

Nearly all the research conducted in this topic shows the use of only a single polymer (melt or solution) spun uniaxially. This section deals with other types of electrospinning involving more than one polymer.

In 2014, Li et al. [47] explored this phenomenon in coaxial electrospinning. Their research agreed with Chakraborty et al. [48] that horizontal electrospinning can reduce imperfections in electrospun scaffolds. They also reported that the Taylor cone may be distorted by the gravitational force in the horizontal setup. On performing further experiments, it was revealed that it was in fact more advantageous to use the vertical setup in case of coaxial spinning as the core–shell structure is not preserved in horizontal setups due to gravity-induced Taylor cone distortion.

Most recently, a study was conducted on blend electrospinning of PCL and gelatin [69], investigating the effects of setup orientation on fibre diameter, pore size, and their respective distributions on flat fibre mats. Solutions of different polymer ratios were electrospun, and it was observed that horizontal electrospinning resulted in a homogeneous fibre diameter distribution. Top-down electrospinning, above a critical concentration of gelatin, resulted in an extremely large fibre diameter distribution. They observed no statistically significant variations in either spinning orientation both in pure PCL and in blend solutions with a low concentration of gelatin.

The associated PhD thesis by Suresh [28] provides a hypothesis for the sudden appearance of extreme heterogeneity when the blend is spun in the top-down orientation with a high gelatin concentration. The thesis explained that viscous stress reversal of the polymer jet described by Taylor in 1969 [6] occurs in both orientations but in different ways. The aerodynamic drag in the horizontal orientation is only balanced out by the electric field force. However, the drag is balanced out by gravity as well as the electric field force in the top-down orientation. This is the reason for slight differences in electrospinning parameters (voltage, TCD, etc.) between both orientations. A marginal discrepancy in spinning parameters was observed by Rodoplu and Mutlu as well [45].

In this particular context of blend electrospinning, the following were thought to contribute to this final effect—the polyelectrolytic nature of gelatin, charge density fluctuations at the Taylor cone, and the oscillation range of voltages. Spinning in both orientations also showed small differences regarding where on the jet the bending instabilities began. In the horizontal setup, the start point of jet instability was seen to move back and forth over a small distance. In the top-down setup, the start point of jet instability fluctuated over a considerable distance.

**Figure 4:**
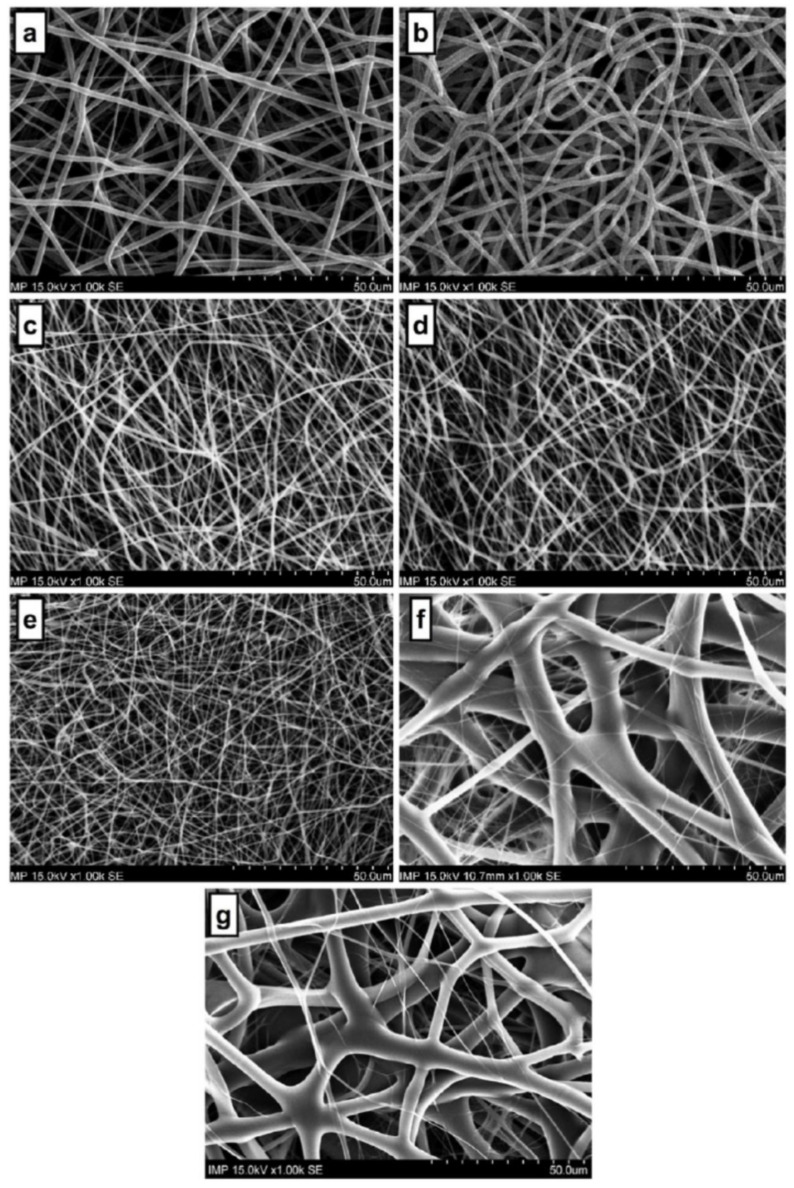
SEM images of PCL and PCL/gelatin electrospun mats spun in horizontal and vertical setup orientations. (Image from [69] reproduced with permission from SAGE Publishing). (**a**) 175 mg/mL PCL (horizontal). (**b**) 175 mg/mL PCL (vertical). (**c**) 150 mg/mL PCL—25 mg/mL gelatin (horizontal). (**d**) 150 mg/mL PCL—25 mg/mL gelatin (vertical). (**e**) 125 mg/mL PCL—50 mg/mL gelatin (horizontal). (**f**) 125 mg/mL PCL—50 mg/mL gelatin (vertical). (**g**) 100 mg/mL PCL—75 mg/mL gelatin (vertical). Note: 100 mg/mL PCL—75 mg/mL gelatin blend was unspinnable in the horizontal setup orientation.

The only parameter that was different in both setups was the direction of the electric field with respect to gravity. This means that gravity either exacerbated or dampened the combined effect of these aspects, depending on whether it acted in the same direction as the electric field or acted perpendicular to it. For the same blend (125 mg/mL PCL—50 mg/mL gelatin), the vertical setup caused a large fibre diameter distribution with pore sizes about four times than that of its horizontal counterpart (Figure 4).

We can see from this study that while gravity has an effect on the spinning of all the different samples, the final effect of varied fibre diameter is ultimately a function of the polymer used. This effect is only observable because of the peculiar properties of a PCL/gelatin blend having gelatin above a critical concentration. Otherwise, the effect of gravity is not prominently observed. An investigation into the effect of apparatus orientation in blend electrospinning provides a gamut of future possibilities. By changing the ratio of the blend components and using polymers with vastly different characteristics together, we can possibly achieve a range of new scaffold morphologies just by changing the setup orientation.

### 2.8. Centrifugal Electrospinning

We have talked extensively about how the apparatus orientation and gravity can influence different types of electrospinning. But this review would be incomplete if we did not mention a scenario where gravity never has any significant contribution. Centrifugal electrospinning is very interesting because the primary driving force of the jet is the centrifugal force. This driving force is exacerbated when an electric field is applied. Several papers have addressed this topic, and it seems to be the only area where there is a consensus—regardless of whether it is solution or melt electrospinning, gravity is so weak compared with the centrifugal force that its effects are negligible [70,71,72,73,74,75,76,77,78].

## 3. Discussion

Interlaboratory inconsistencies are expected to an extent in electrospinning because each experiment is unique in terms of materials used, equipment configuration, and environmental conditions. It is difficult to ascertain if the discrepancies arise from expected variations between labs or if they arise from basic differences in the experiment itself. For example, given the same experimental conditions, would there be similar results for average reported fibre diameter between Yang et al. (who used solution electrospinning) and Wunner et al. (who used melt electrospinning)? On a similar note, why did Yang et al. and Khenoussi et al. have different results for fibre diameter distribution when they both used solution electrospinning? Is it because they used different polymers, or is it because of random uncontrollable factors that vary with each lab? And why did Rodoplu and Mutlu see no more than Taylor cone distortion and parabolic jets in the horizontally oriented system? Had they used PTFE or PAN instead of PVA, would their results have aligned with Yang et al. or Khenoussi et al., respectively?

Nevertheless, despite the sometimes-conflicting results across the literature, it is clear (from the limited number of papers that address this issue) that there is some effect of gravity on the electrospinning process. Yes, the effect of gravity is a lot smaller than the effect of the electric field. But empirical observations suggest that despite this difference in magnitude, gravity does influence the final product. Yet it is very rarely the main topic of study. Even when addressed, it is usually mentioned as a secondary observation.

It is important that each researcher choose the setup best suited to their application. Any number of variables can factor into this decision. A key example would be the choice of polymer. Take, for instance, PCL. PCL requires a large tip-to-collector distance for stable spinning. In this case, a researcher may opt for a vertical setup to avoid the parabolic jet that is guaranteed to appear in a horizontal setup at this distance. A horizontal setup may be preferred when using poly(ethylene oxide) (PEO), where a short tip-to-collector distance is recommended. In this case, the effect of gravity on the jet trajectory is lesser, and the final product may have less artefacts. Another important factor is the flow rate, as demonstrated by several researchers, especially Wunner et al. The effect of gravity is not significant when the flow rate is low. Therefore, while building specialised electrospinning setups for specific applications, it would be prudent to consider how different parameters can subdue or exacerbate the effect of gravity on the spinning process.

## 4. Conclusions

With this review, we present a definite need for further research into the effect of setup orientation in electrospinning. In addition, there is a need to further investigate how blended polymer solutions or doped polymer solutions will behave in either orientation. Considering the differences in physical and chemical properties between the constituents of the blend, the spinning in vertical or horizontal orientation can be vastly different. It would also be compelling to see if the microstructure (fibre diameter and pore size distributions) of scaffolds can somehow be modified or guided by the angle of the electrospinning setup. If the microstructure could be easily manipulated that way, it would completely change (and simplify) the way the electrospinning community attempts to create mixed and multilayered scaffolds.

This review shows us that it is important to choose the electrospinning orientation carefully according to the objectives of each individual experiment. We have provided an exhaustive account of the minimal literature available on this topic. Depending on stipulated priorities, researchers can now use this review to identify the best setup for their research initiatives.

## Figures and Tables

**Table 1:** Electrospinning parameters and their effects.

**Process Parameters**	**Increase**	**Decrease**
Voltage [4,8,10,11] 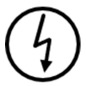	- Smaller Taylor cone, thinner fibres, smaller pores. - Very high voltage causes multiple jets and may result in the formation of beads.	- Larger Taylor cone, thicker fibres, larger pores. - Voltage below spinning threshold stops jet formation.
Flow rate [4,8,10,12] 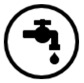	- Thicker fibres, larger pores. - Excessive flow rate causes a bloated Taylor cone and wet fibres.	- Thinner fibres, smaller pores - Too little causes the Taylor cone to retreat into the nozzle.
Tip-to-collector distance (TCD) [4,10,13] 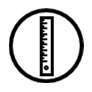	- Causes an effective decrease in electrical density, resulting in thicker fibres. - A large TCD may not be suitable for solvents with fast evaporation.	- Decrease in TCD causes an effective increase in electrical density. - A minimum distance is required for the formation of fibres.
**Solution Parameters**	**Increase**	**Decrease**
Polymer concentration [4,10,14] 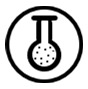	- Thicker fibres, larger pores. - Very high concentration stops electrospinning. - A medium concentration gives a combination of beads and fibres.	- Thinner fibres, smaller pores. - Very low concentration results in the formation of beads. - If the polymer is sufficiently conductive, electrospraying is possible at fairly low concentrations.
Conductivity [4,10,15] 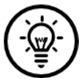	- Thinner fibres, smaller pores. - Solutions with extremely high conductivities may be unstable during electrospinning and are likely to produce multiple jets.	- Thicker fibres, larger pores produced. - A minimum conductivity is required for electrospinning.
Viscosity [4,14,16] 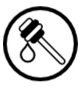	- Thicker fibres, larger pores - Extremely viscous solutions cannot be electrospun.	- Thinner fibres, smaller pores - Very low viscosity causes the formation of beads.
Surface tension [4,14,17,18] 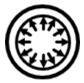	- Makes it harder to electrospin and results in instability of jets. - Solutions with very high surface tension cannot be electrospun.	- Very low surface tension increases the tendency of jet breakage and results in drops.
Molecular weight [4,11,15] 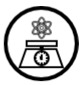	- Beads reduced if any, thicker fibres produced.	- Fibre thickness decreases. - Very low molecular weight forms beads.
**Ambient Parameters**	**Increase**	**Decrease**
Temperature [4,10,19] 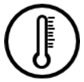	- Fibre diameter decreases.	- Fibre diameter increases.
Humidity [4,10,19,20,21,22] 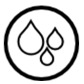	- Higher humidity causes thinner fibres due to slower solvent evaporation. - Increases the incidence and size of circular pores on fibres. - No useable fibres produced over a critical limit	- Lower humidity allows for better and faster solvent evaporation, resulting in thicker fibres.
**Other Parameters**	**Effect**	
Total spinning time 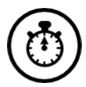	- Longer spinning times result in the accumulation of ions in the vicinity of the jet, resulting in instabilities in the process.	
Scaffold thickness 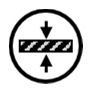	- Very thick scaffolds can induce impedance, causing fibres to be deflected off the target. - Extremely thin samples can be easily electrospun, but it may not be feasible to remove these samples properly from the collector or process them for further testing.	
Solvent vapour partial pressure [10,23] 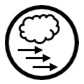	- Solvents with high vapour partial pressure evaporate faster and tend to produce thinner fibres.	
Polymer relaxation time [24] 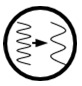	- Polymer relaxation time needs to be above a particular threshold for the polymer to be spinnable. - If the polymer relaxation time is sufficiently large, electrospinnability may be improved even with dilute polymer solutions.	
Relative collector velocity [25,26] 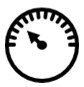	- Increase in this velocity results in a lower fibre diameter and degree of orientation distribution up to a critical velocity.	
Spinning orientation	- Effects discussed in this paper.	
